# Somatostatin Analogues Do Not Improve Survival in Grade 3 Gastroenteropancreatic Neuroendocrine Neoplasms: A Registry-Based Analysis with Ki-67 Stratification

**DOI:** 10.3390/cancers18203357

**Published:** 2026-10-17

**Authors:** Alexander R. Siebenhüner, Jean-Benoît Rossel, Emanuel Christ, Attila Kollár

**Affiliations:** 1 Department of Gastroenterology and Hepatology, University Hospital Zurich, University of Zurich, 8091 Zurich, Switzerland; 2 Department of Hematology and Oncology, Hirslanden Klinik Zurich, 8032 Zurich, Switzerland; 3 Department of Clinical Research, University of Bern, 3010 Bern, Switzerland; 4 Clinic for Radiology and Nuclear Medicine, Center for Neuroendocrine and Endocrine Tumors, University Hospital Basel, 4031 Basel, Switzerland; 5 ENETS Center of Excellence, University Hospital Basel, 4031 Basel, Switzerland; 6 Center of Endocrine and Neuroendocrine Tumors, Department of Endocrinology, Diabetes and Metabolism, University Hospital Basel, University of Basel, 4031 Basel, Switzerland; 7 Department of Medical Oncology, Inselspital, Bern University Hospital, University of Bern, 3010 Bern, Switzerland; attila.kollar@insel.ch

**Keywords:** neuroendocrine neoplasm, somatostatin analogues, grade 3, Ki-67, NET G3, neuroendocrine carcinoma, survival analysis, gastroenteropancreatic, registry

## Abstract

Background/Objectives: The efficacy of somatostatin analogues (SSA) in Grade 3 (G3) neuroendocrine neoplasms (NEN) remains controversial, and registry data rarely permit a formal separation of NET G3 from neuroendocrine carcinoma (NEC). Methods: We analyzed 519 patients with G3 gastroenteropancreatic (GEP) NEN recorded in a national neuroendocrine tumor registry (data export 3 April 2025; 464 de novo G3, 55 transformed G3); bronchopulmonary primaries were excluded. A Ki-67 > 55%, entered as a time-varying indicator, served as a surrogate for NEC. Outcomes were compared using Kaplan–Meier and Cox proportional hazards models with all treatments and the Ki-67 > 55% indicator entered as time-varying covariates. Results: Among 410 patients with classifiable Ki-67, 256 (62.4%) ever had a Ki-67 > 55%. Ki-67 > 55% was associated with worse progression-free survival (PFS; HR 1.40, 95% CI 1.06–1.84; *p* = 0.016) and overall survival (OS; HR 1.65, 95% CI 1.15–2.37; *p* = 0.006). SSA use was not significantly associated with PFS (HR 1.32, 95% CI 0.85–2.04; *p* = 0.215) or OS (HR 0.91, 95% CI 0.54–1.56; *p* = 0.744). After adjustment for Ki-67, transformed G3 was associated with worse OS (HR 1.60, 95% CI 1.02–2.51; *p* = 0.040). Conclusions: SSA therapy was not associated with improved survival in G3 GEP-NEN. As this is an observational analysis subject to confounding by indication, these estimates do not establish a lack of biological efficacy of SSA. A Ki-67 threshold of 55% identified a prognostically distinct subgroup and explains why transformed G3 appears indolent: after adjustment, it was associated with worse overall survival. This finding rests on 55 patients with 29 deaths and is hypothesis-generating.

## Simple Summary

Neuroendocrine neoplasms are rare tumors that may grow slowly or very fast. In the slow-growing forms, drugs called somatostatin analogues are a standard treatment, and doctors often continue them once a tumor becomes fast-growing, particularly in patients who did well on them before. Whether this helps is unknown. We studied 519 patients with fast-growing neuroendocrine tumors of the digestive tract and pancreas recorded in a national registry. Patients who received somatostatin analogues did not survive longer and their disease did not stay stable for longer, once we accounted for the fact that they were younger and had slower-dividing tumors to begin with. How fast the tumor cells divided predicted outcome far better. Our findings suggest that these drugs should be given to control symptoms or on other specific grounds, rather than simply because a patient received them earlier in the disease.

## 1. Introduction

Neuroendocrine neoplasms (NENs) constitute a heterogeneous group of tumors arising from the diffuse neuroendocrine system with varying clinical behavior, biological aggressiveness, and treatment responses [1]. The 2017/2019 World Health Organization (WHO) classification represents a significant advancement in the understanding of these tumors, particularly with the introduction of a revised grading system that differentiates high-grade (G3) NENs into two distinct biological entities: well-differentiated neuroendocrine tumors grade 3 (NET G3) and poorly differentiated neuroendocrine carcinomas (NEC) [2,3,4,5].

This classification acknowledges the heterogeneity within G3 NEN, which are defined by a proliferation rate above 20% (Ki-67 > 20% and/or >20 mitoses per 10 high-power fields). NET G3 maintains the architectural and cytological features of well-differentiated tumors despite elevated proliferation indices, while NEC exhibits poorly differentiated morphology with high-grade cytological features [6]. This distinction has critical therapeutic implications, as these entities differ substantially in their biological behavior, molecular alterations, and response to various treatment modalities. It is, however, a morphological distinction that requires review of the primary material and that entered routine digestive pathology only with the 2019 WHO classification, consolidated in the 2020 IARC framework. Cohorts assembled over longer periods therefore contain a substantial proportion of cases for which the distinction was never formally made. A consensus re-evaluation of high-grade gastroenteropancreatic NEN (GEP-NEN) has since defined the morphological criteria that best separate the two entities and shown that they carry prognostic weight [7].

A particularly intriguing subgroup within NET G3 comprises tumors that evolve from previously lower-grade (G1/G2) neuroendocrine tumors, termed “transformed G3 NETs.” These transformed tumors may retain some biological characteristics of their lower-grade predecessors, potentially influencing their clinical course and therapeutic responsiveness [8]. Recent molecular studies have demonstrated that progression from low-grade NET to high-grade neoplasms can occur via the acquisition of TP53 and RB1 alterations, similar to NEC, though the clinical implications remain under investigation [9,10]. The recognition of transformed G3 NETs as a distinct clinical entity has emerged from observations that these tumors often demonstrate a more indolent course compared to de novo G3 NEN, with implications for treatment selection and sequencing [11].

Somatostatin analogues (SSAs) represent a cornerstone in the management of well-differentiated G1/G2 NETs, with proven antisecretory and antiproliferative efficacy established in landmark trials such as PROMID and CLARINET [12,13]. Dose escalation has additionally been explored in progressive lower-grade disease [14]. Their role in G3 NEN, however, remains controversial. Current European Neuroendocrine Tumor Society (ENETS) guidance recommends SSAs primarily for functioning NET G3 with relatively low Ki-67 (<55%), but their role as monotherapy in non-functioning high-grade tumors is not supported by current evidence [15]. Recently, a subgroup analysis of the NETTER-2 trial reported a response rate of 7.4% and a median progression-free survival (PFS) of 5.6 months for high-dose SSA in the control arm [16,17]. Consequently, peptide receptor radionuclide therapy (PRRT) and systemic chemotherapy (capecitabine/temozolomide) are currently recommended as first-line treatment for advanced gastroenteropancreatic NET G3, with platinum-based regimens recommended for NEC [15,16,18]. The evidence for PRRT in high-grade disease has been summarized separately [19]. Outcomes in advanced high-grade digestive NEN remain poor, as recently reported in the prospective NORDIC NEC 2 cohort of 639 patients [20].

Despite these recommendations, the potential benefit of SSAs in the subset of transformed G3 NETs—which may retain somatostatin receptor expression like their lower-grade counterparts—warrants further investigation. The biological continuum between G1/G2 NETs and transformed G3 NETs suggests that therapeutic approaches, effective in lower-grade tumors, might maintain activity in this specific subpopulation [21].

This study addresses this knowledge gap by analyzing the association between SSA use and survival in a large cohort of patients with G3 gastroenteropancreatic NEN from the Swiss national neuroendocrine tumor registry (SwissNET). Because centralized morphological review was not available, we used a Ki-67 threshold of 55% as a surrogate for the NET G3/NEC distinction and treated this stratification as the primary axis of the analysis. The comparison between de novo and transformed G3 tumors is reported as a secondary question.

## 2. Materials and Methods

### 2.1. Data Source and Patient Population

Data for this study were extracted from the Swiss national neuroendocrine tumor registry (SwissNET) on 3 April 2025. The registry is a nationwide database that has prospectively collected comprehensive clinical, pathological, and outcome data for patients with neuroendocrine neoplasms since 2009. All patients whose tumors were classified as G3 (Ki-67 > 20% and/or mitotic count > 20 per 10 high-power fields) were included in the analysis. Because the NET G3 category is not established for bronchopulmonary neuroendocrine neoplasms, patients with a lung primary (*n* = 36) were excluded, leaving a gastroenteropancreatic cohort of 519 patients (Supplementary Figure S1). For each patient, baseline was defined as the date on which the tumor was first classified as G3. Patients were categorized into two main groups: (1) those with de novo G3 tumors at initial diagnosis, and (2) those with transformed G3 tumors that were previously classified as G1 or G2. The study was approved by the responsible cantonal ethics committee, and the registry is listed on ClinicalTrials.gov; approval and registration details are given on the title page. All participants provided written informed consent in accordance with the Declaration of Helsinki.

### 2.2. Variable Definitions and Data Collection

Demographic and clinical characteristics collected included age at diagnosis, sex, primary tumor site, functional status, tumor size and Ki-67 proliferation index. Treatment variables included the use of SSAs (lanreotide, octreotide LAR, subcutaneous octreotide or pasireotide), chemotherapy, and molecular targeted therapies, defined as binary variables that varied over time. Surgery, ablative therapy and peptide receptor radionuclide therapy (PRRT) are recorded as single dated events (punctual therapies) and were counted from baseline. The registry records radionuclide and external beam treatments under a single irradiation-therapy item; PRRT accounts for approximately 70–75% of these treatments, the remainder being external beam radiotherapy. This covariate is referred to as PRRT throughout and should be interpreted with that composition in mind.

### 2.3. Ki-67 Stratification

Centralized morphological second review of archival material was not feasible, and the WHO distinction between NET G3 and NEC was formalized for digestive tumors only in 2019 and consolidated in 2020, whereas registry recruitment started in 2009. We therefore used the proliferation index as a surrogate. A Ki-67 threshold of 55% was first identified in the NORDIC NEC cohort as the cut-off that best separated response and survival within high-grade disease, and it has been used repeatedly for this purpose since [7,22]. Patients with a documented Ki-67 > 55% at any time point were classified as NEC-like; patients whose Ki-67 never exceeded 55% were classified as NET G3-like. Because Ki-67 was reassessed during follow-up in a substantial number of patients, this indicator was modeled as a time-varying covariate. Patients without any classifiable Ki-67 value (*n* = 109, 21.0%) were not assigned to either category, leaving 410 patients for the Ki-67-stratified analyses. This surrogate cannot capture cases in which morphology and proliferation index are discordant and is not a substitute for integrated morphological assessment. In high-grade GEP-NEN, proliferation index and morphology do not coincide in every case: a proportion of well-differentiated tumors exceeds a Ki-67 of 55%, and a proportion of poorly differentiated carcinomas falls below it [7,11]. The 55% threshold was therefore used as a pragmatic, pre-specified surrogate and not as a diagnostic criterion. Patients without a classifiable Ki-67 value were handled using a complete-case analysis in the Ki-67-stratified models; no imputation and no sensitivity analysis for missing Ki-67 values were performed. Their baseline characteristics were compared with those of patients with a classifiable value (Supplementary Table S1).

### 2.4. Outcome Measures

The primary outcomes were progression-free survival (PFS) and overall survival (OS). For PFS, each patient was followed until the last visit (censoring), for a maximum of 5 years, or until disease progression, relapse, or death (events). Disease progression was assessed based on clinical documentation in the registry, including radiological progression where available or initiation of a new treatment modality as a proxy for real-world progression. For OS, only death was considered an event.

### 2.5. Statistical Analysis

Categorical variables were described with frequencies and percentages of the total group size, with missing values reported as a separate category and excluded from the statistical tests; they were compared between groups using Fisher’s exact tests. Continuous variables were presented as median and interquartile range (IQR) and compared using Mann–Whitney–Wilcoxon tests. Survival probabilities were estimated using the Kaplan–Meier method, and differences between groups were assessed with log-rank tests. Multivariable Cox proportional hazards models were used to evaluate the association between SSA treatment and survival outcomes, adjusting for age, sex, prior G1/G2 status and concomitant therapies. The use of SSA, chemotherapy and molecular therapy and the cumulative numbers of surgeries, ablative therapies and PRRT treatments were modeled as time-varying covariates. The main model additionally included the time-varying Ki-67 > 55% indicator. Treating exposures that begin after baseline as time-varying avoids the immortal-time bias that arises when they are handled as fixed [23,24]. Counts of punctual therapies were capped at 2 surgical procedures, 1 ablative therapy and 4 PRRT treatments to avoid sparsely populated categories and were entered as ordinal terms; their distribution is given in Supplementary Table S2. SSA use, chemotherapy and molecular therapy were coded as binary time-varying indicators that took the value 1 between the documented start and end dates of the respective therapy and 0 otherwise; patients with ongoing SSA at baseline entered the model with the indicator set to 1. The Ki-67 > 55% indicator took the value 1 from the date of a documented Ki-67 value above 55% and returned to 0 if a subsequent value was 55% or lower. The data were arranged with one record per patient for each interval between changes in covariate status or events, so that exposure status at any time point reflected only information available up to that time point, and Cox models were fitted with the stcox command. Somatostatin receptor status, tumor size and laboratory values were not included because of the proportion of missing values. Two sensitivity analyses were pre-specified: exclusion of patients who had started SSA before baseline, and restriction to patients with documented metastatic disease. Confounding by indication was addressed in three ways: by modeling treatments as time-varying exposures, by adjusting for age, sex, prior G1/G2 status, concomitant therapies and the Ki-67 > 55% indicator, and by the sensitivity analysis that excluded the 23 patients who had started SSA before baseline. Performance status, tumor burden and somatostatin receptor expression were not available or incompletely documented in the registry; residual confounding by these factors cannot be excluded, and all estimates are reported as associations. Heterogeneity of the association between SSA use and survival was explored with interaction terms for primary site (pancreatic vs. non-pancreatic), de novo vs. transformed G3, and a documented Ki-67 > 55% at any time before baseline (yes vs. no), in the main model including the time-varying Ki-67 indicator; the association of chemotherapy with overall survival was examined in the same way. The proportional hazards assumption for transformed G3 was assessed with tests based on Schoenfeld residuals. Kaplan–Meier curves are presented with numbers at risk. Kaplan–Meier curves by Ki-67 category were drawn with time-varying group membership, so that each patient contributed to the NET G3-like or NEC-like curve according to the Ki-67 category at each time point, and the curves were compared with the log-rank test extended to time-dependent group membership (Mantel–Byar test). All statistical analyses were performed using Stata version 19.0 (StataCorp, College Station, TX, USA); the statistical report was produced with R version 4.5.1 (R Foundation for Statistical Computing, Vienna, Austria). *P*-values less than 0.05 were considered statistically significant.

## 3. Results

### 3.1. Patient Characteristics

Patient characteristics are summarized in Table 1. A total of 519 patients with G3 GEP-NEN were included, comprising 464 (89.4%) with de novo G3 tumors and 55 (10.6%) with transformed G3 tumors. Patients with transformed G3 were younger at diagnosis than those with de novo G3 (median age 61.1 vs. 69.3 years, *p* < 0.001) and had lower Ki-67 indices at baseline (median 34.5% vs. 70.0%, *p* < 0.001). Pancreatic primaries (49.1% vs. 21.3%) and ileal or jejunal primaries (12.7% vs. 3.9%) were more frequent in the transformed G3 group (*p* < 0.001 for primary site), as were functioning tumors (10.9% vs. 4.1%, *p* = 0.033). A Ki-67 > 55% was documented at least once in 21.8% of transformed and 52.6% of de novo G3 patients (*p* < 0.001), with Ki-67 missing in 9.1% and 22.4%, respectively. SSA treatment was documented in 49.1% of transformed and 9.7% of de novo G3 patients (*p* < 0.001).

### 3.2. Characteristics of SSA-Treated Patients

Overall, 72 patients (13.9%) received SSA at some point during their disease course (Table 2). SSA-treated patients were younger at diagnosis (median age 59.5 vs. 69.8 years, *p* < 0.001), more often had pancreatic primaries (55.6% vs. 19.2%, *p* < 0.001 for primary site) and functioning tumors (15.3% vs. 3.1%, *p* < 0.001), had larger tumors at baseline (median 40.0 vs. 20.0 mm, *p* = 0.014) and lower Ki-67 indices (median 30.0% vs. 70.0%, *p* < 0.001), and more frequently had prior G1/G2 grading (37.5% vs. 6.3%, *p* < 0.001). Twenty-three of the 72 SSA-treated patients (31.9%) had started SSA before the first documentation of G3, predominantly in the transformed group (63.0% vs. 13.3%, *p* < 0.001). Among SSA-treated patients, median observation time was shorter in the transformed than in the de novo group (1.1 vs. 3.0 years, *p* = 0.042). Follow-up, treatment sequence and liver metastases among SSA-treated patients are detailed in Supplementary Table S3. Among the 72 SSA-treated patients, 40 (55.6%) ever received lanreotide, 33 (45.8%) octreotide LAR, nine (12.5%) subcutaneous octreotide and one (1.4%) pasireotide, and several patients switched between substance or formulation during treatment. The median duration of SSA treatment was 0.8 years (IQR 0.2–1.9), and the dosage was documented for 12 patients (16.7%) (Supplementary Table S4).

### 3.3. Characteristics by Ki-67 Category

Ki-67 values permitting classification were available for 410 patients (79.0%; Supplementary Table S5). Of these, 256 (62.4%) had a Ki-67 > 55% at least once and 154 (37.6%) never exceeded this threshold. Patients in the Ki-67 > 55% group were older at diagnosis (median 69.4 vs. 63.3 years, *p* < 0.001) and more often male (65.2% vs. 47.4%, *p* < 0.001), less frequently had pancreatic primaries (14.1% vs. 50.0%, *p* < 0.001 for primary site), less frequently had prior G1/G2 grading (4.7% vs. 24.7%, *p* < 0.001), and less frequently received SSA (6.2% vs. 29.9%, *p* < 0.001). Compared with the 410 patients with a classifiable value, the 109 patients without a classifiable Ki-67 value were older (median 71.8 vs. 67.5 years, *p* = 0.015), less frequently had pancreatic primaries (11.9% vs. 27.6%, *p* = 0.002 for primary site) and less frequently had transformed G3 (4.6% vs. 12.2%, *p* = 0.022); the proportion of SSA-treated patients did not differ significantly (9.2% vs. 15.1%, *p* = 0.121) (Supplementary Table S1).

### 3.4. Survival Outcomes

#### 3.4.1. Univariable Analysis

In the overall cohort, 314 patients (60.5%) experienced disease progression, relapse, or death and 191 (36.8%) died. Event proportions were higher in the transformed than in the de novo G3 group (74.5% vs. 58.8%, *p* = 0.028; death 52.7% vs. 34.9%, *p* = 0.012; Supplementary Table S6) and higher in SSA-treated than in untreated patients (88.9% vs. 55.9%, *p* < 0.001; death 55.6% vs. 33.8%, *p* < 0.001; Supplementary Table S7). Staging information was available for 236 patients (45.5%), of whom 107 had metastatic disease; event proportions were higher in metastatic than in localized disease (83.2% vs. 51.2%, *p* < 0.001; death 46.7% vs. 27.9%, *p* = 0.004; Supplementary Table S8).

**Figure 1:**
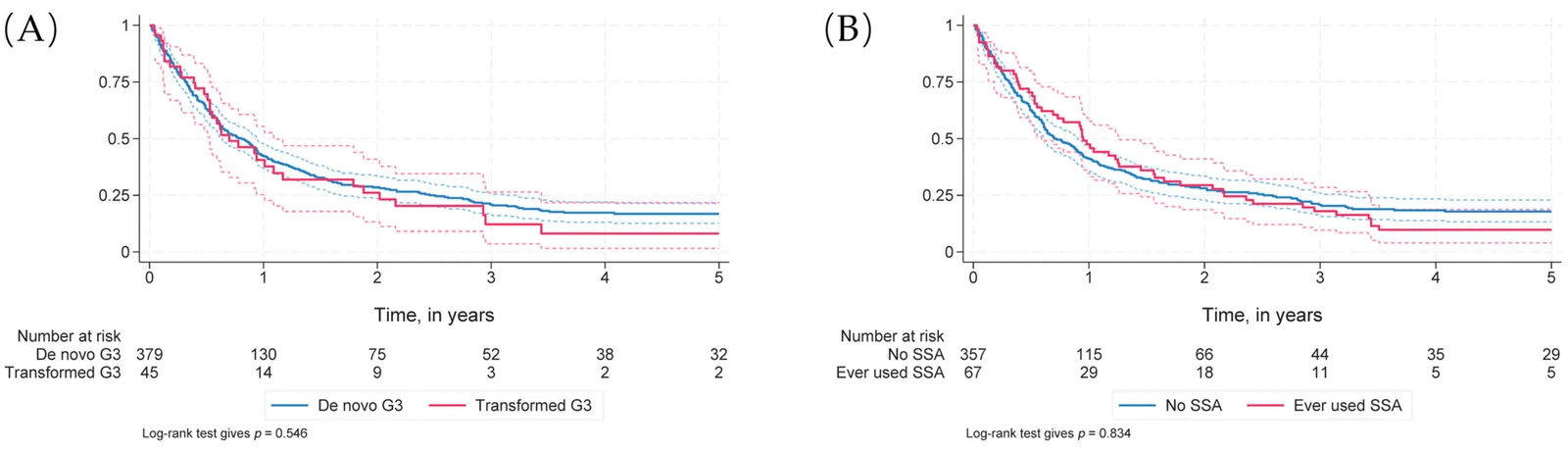
Kaplan–Meier curves for progression-free survival. (**A**) By transformation status (de novo vs. transformed G3), log-rank *p* = 0.546. (**B**) By use of somatostatin analogues (SSA), log-rank *p* = 0.834. Numbers at risk are shown below each panel. Solid lines show Kaplan–Meier estimates and dashed lines the 95% confidence intervals; blue lines, de novo G3 (**A**) and no SSA (**B**); red lines, transformed G3 (**A**) and ever used SSA (**B**). G3, grade 3; SSA, somatostatin analogue.

**Figure 2:**
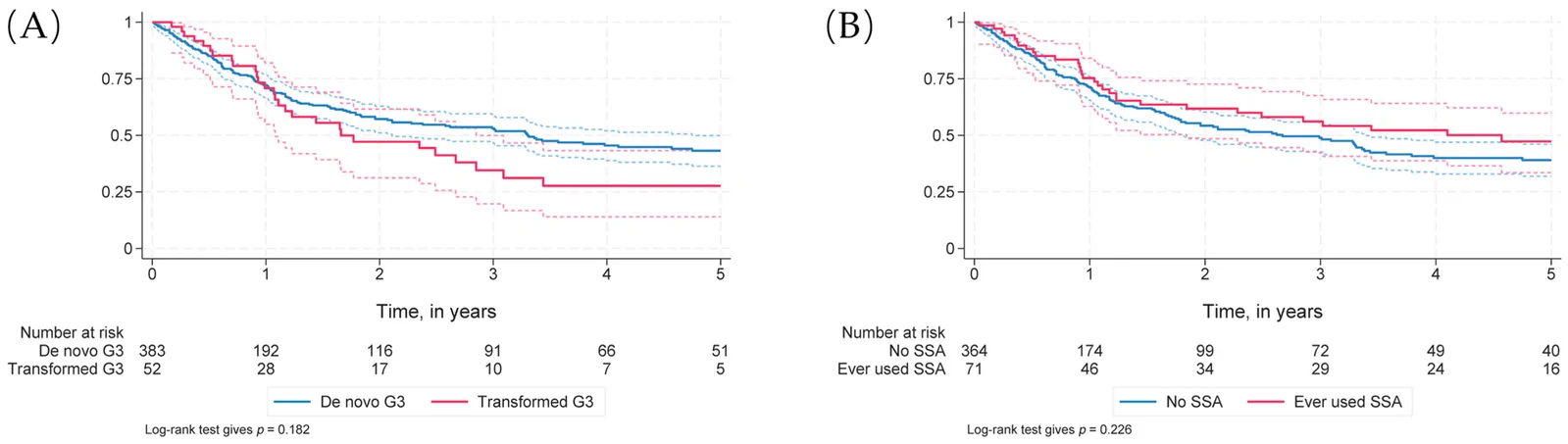
Kaplan–Meier curves for overall survival. (**A**) By transformation status, log-rank *p* = 0.182. (**B**) By use of SSA, log-rank *p* = 0.226. Numbers at risk are shown below each panel. Solid lines show Kaplan–Meier estimates and dashed lines the 95% confidence intervals; blue lines, de novo G3 (**A**) and no SSA (**B**); red lines, transformed G3 (**A**) and ever used SSA (**B**). G3, grade 3; SSA, somatostatin analogue.

**Figure 3:**
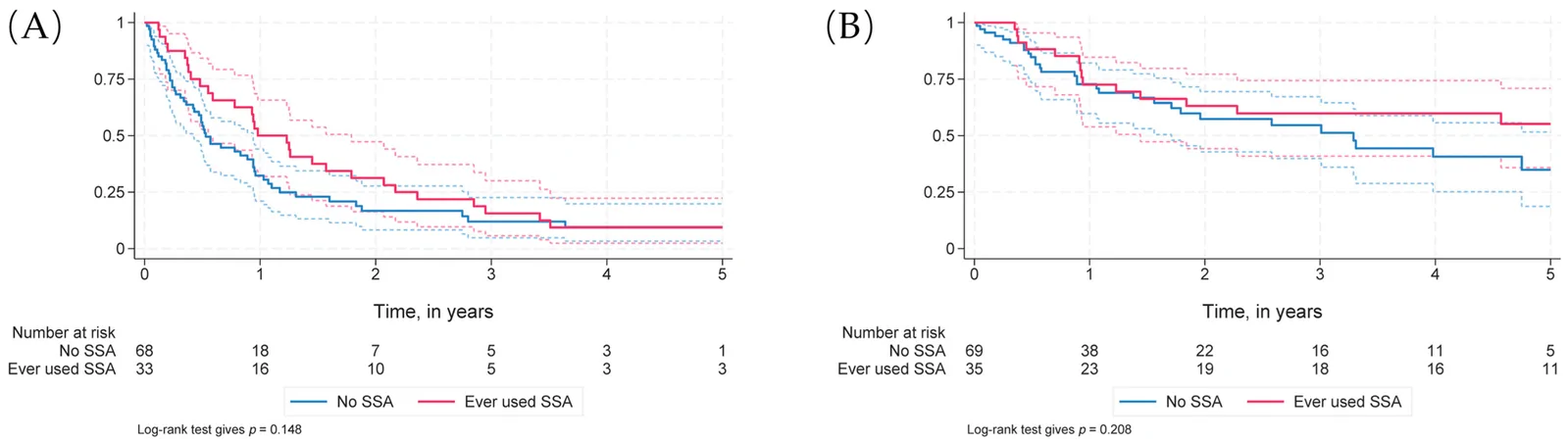
Kaplan–Meier curves in the metastatic subgroup (*n* = 107), by use of SSA. (**A**) Progression-free survival, log-rank *p* = 0.148. (**B**) Overall survival, log-rank *p* = 0.208. Numbers at risk are shown below each panel. Solid lines show Kaplan–Meier estimates and dashed lines the 95% confidence intervals; blue lines, no SSA; red lines, ever used SSA. G3, grade 3; SSA, somatostatin analogue.

**Figure 4:**
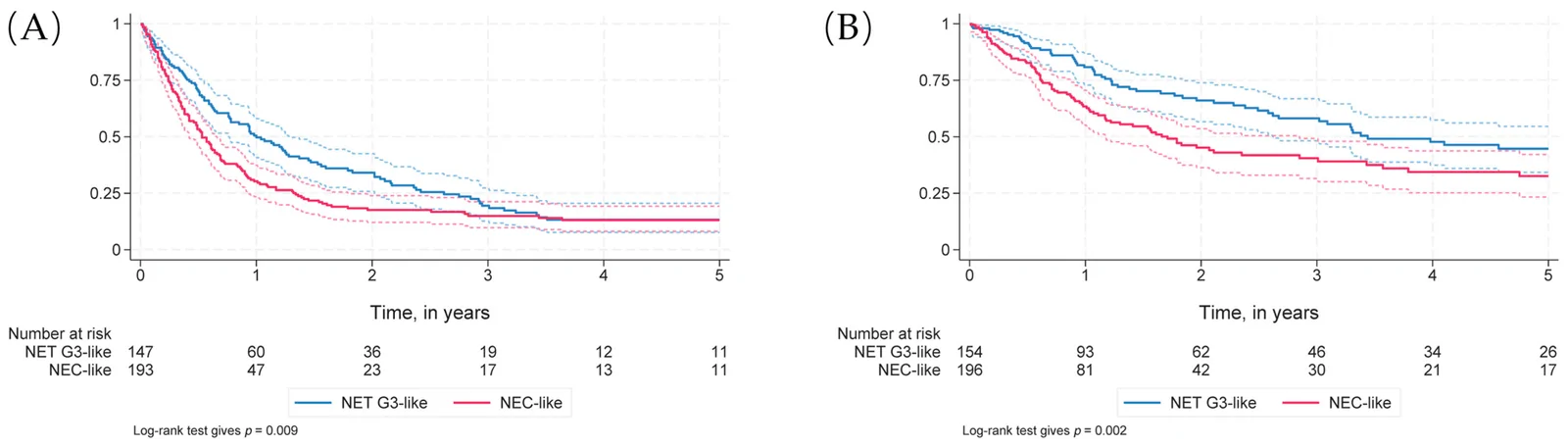
Kaplan–Meier curves by Ki-67 category (NET G3-like vs. NEC-like) with time-varying group membership: each patient contributes to the curve corresponding to the Ki-67 category at each time point. (**A**) Progression-free survival, *p* = 0.009. (**B**) Overall survival, *p* = 0.002. *p*-values from the log-rank test extended to time-dependent group membership. Numbers at risk are shown below each panel. Solid lines show survival estimates and dashed lines the 95% confidence intervals; blue lines, NET G3-like; red lines, NEC-like.

These crude proportions were not reflected in the time-to-event analyses. Log-rank tests showed no significant differences between de novo and transformed G3 (PFS *p* = 0.546; OS *p* = 0.182) or between SSA-treated and untreated patients (PFS *p* = 0.834; OS *p* = 0.226; Figure 1 and Figure 2), and the same applied within the metastatic subgroup (PFS *p* = 0.148; OS *p* = 0.208; Figure 3). The discrepancy is accounted for by the shorter observation time in the transformed G3 and SSA-treated groups: events are concentrated within a shorter follow-up window, which raises the crude proportion without implying a higher event rate per unit of time. Kaplan–Meier curves by Ki-67 category (NET G3-like vs. NEC-like) are shown in Figure 4; NEC-like status was associated with shorter PFS (*p* = 0.009) and OS (*p* = 0.002).

#### 3.4.2. Multivariable Analysis

In the multivariable Cox proportional hazards model adjusted for age, sex and therapies, but without the Ki-67 indicator, SSA use was not significantly associated with PFS (HR 1.46, 95% CI 0.98–2.17; *p* = 0.060) or OS (HR 0.98, 95% CI 0.60–1.61; *p* = 0.945), and transformed G3 was associated with neither PFS (HR 1.18, 95% CI 0.81–1.71; *p* = 0.396) nor OS (HR 1.51, 95% CI 0.98–2.32; *p* = 0.060) (Supplementary Tables S9 and S10).

In the main model including the time-varying Ki-67 > 55% indicator (*n* = 410; Table 3), Ki-67 > 55% was associated with worse PFS (HR 1.40, 95% CI 1.06–1.84; *p* = 0.016) and worse OS (HR 1.65, 95% CI 1.15–2.37; *p* = 0.006). SSA use remained non-significant for both endpoints (PFS HR 1.32, 95% CI 0.85–2.04, *p* = 0.215; OS HR 0.91, 95% CI 0.54–1.56, *p* = 0.744). After adjustment for Ki-67, transformed G3 was associated with worse OS (HR 1.60, 95% CI 1.02–2.51; *p* = 0.040) but not with PFS (HR 1.11, 95% CI 0.75–1.63; *p* = 0.609). Tests based on Schoenfeld residuals did not indicate a violation of the proportional hazards assumption for transformed G3 (PFS *p* = 0.647; OS *p* = 0.124). Chemotherapy was associated with better OS (HR 0.47, 95% CI 0.28–0.79; *p* = 0.005). Within subgroups, this association was consistent, with no significant interaction by primary site (*p* = 0.202), transformation status (*p* = 0.479) or Ki-67 category (*p* = 0.352) (Supplementary Table S11, Supplementary Figure S2). A dose–response analysis was not possible because substances, dosages and schedules of chemotherapy were documented heterogeneously in the registry. A higher cumulative number of surgical procedures was associated with better PFS (HR 0.58, 95% CI 0.46–0.74; *p* < 0.001) and OS (HR 0.48, 95% CI 0.35–0.66; *p* < 0.001), and molecular therapy with better PFS (HR 0.32, 95% CI 0.12–0.89; *p* = 0.028); ablative therapy and PRRT were not significantly associated with either endpoint.

After exclusion of the 23 patients who had started SSA before baseline, SSA use remained non-significant for PFS (HR 1.30, 95% CI 0.82–2.08; *p* = 0.267) and OS (HR 0.76, 95% CI 0.40–1.42; *p* = 0.385) (Supplementary Tables S9 and S10, Supplementary Figures S3 and S4). The association between SSA use and survival was examined across subgroups defined by primary site, de novo vs. transformed G3, and Ki-67 category (Supplementary Table S12, Supplementary Figure S5). In patients without a documented Ki-67 > 55% at any time before baseline, SSA use was associated with worse PFS (HR 2.12, 95% CI 1.27–3.53; *p* = 0.004; interaction *p* = 0.010); no other subgroup-specific association with PFS was significant. For OS, the interaction tests were nominally significant for transformation status (*p* = 0.049) and Ki-67 category (*p* = 0.027), whereas none of the subgroup-specific hazard ratios reached significance.

## 4. Discussion

This analysis of 519 patients with G3 gastroenteropancreatic neuroendocrine neoplasms from the Swiss national neuroendocrine tumor registry (SwissNET) addresses two questions: whether SSA use is associated with survival in G3 disease, and whether a Ki-67 threshold of 55% is a usable surrogate for the NET G3/NEC distinction in registry data. The current data indicate that (1) SSA use was not associated with improved progression-free or overall survival in any model and (2) Ki-67 > 55% was consistently associated with worse outcomes and, once included in the model, altered the interpretation of the de novo versus transformed comparison. Therefore, a Ki-67 threshold of 55% appears to be a usable surrogate for the NET G3/NEC distinction in registry data, although it was not validated against morphology in this cohort.

### 4.1. Biological Heterogeneity and Outcome Within G3 Neuroendocrine Neoplasms

The 2017/2019 WHO classification represented a paradigm shift in the understanding of high-grade neuroendocrine neoplasms by recognizing the existence of well-differentiated NETs with high proliferation rates (NET G3) as distinct from poorly differentiated neuroendocrine carcinomas (NEC) [25,26]. Our study further delineates this heterogeneity by distinguishing between de novo G3 tumors and those that transform from previously lower-grade neoplasms. Patients with transformed G3 tumors demonstrated significantly different characteristics compared to those with de novo G3, including younger age at diagnosis, lower Ki-67 indices, higher prevalence of pancreatic primaries, and a higher prevalence of functioning tumors. The significantly lower median Ki-67 index in transformed G3 tumors is particularly noteworthy, as it positions most of these cases within the NET G3 category rather than NEC according to current conceptual frameworks [27,28]. Recent molecular studies have identified distinct genetic alterations between NET G3 and NEC, with mutations in MEN1, DAXX, and ATRX predominating in the former, and TP53 and RB1 mutations characterizing the latter [29,30]. While the registry does not include molecular profiling, the clinicopathological differences observed between transformed and de novo G3 tumors likely reflect these underlying genetic disparities, with implications for treatment selection and sequencing.

Our data qualify the assumption that transformed G3 tumors follow a more favorable course. Transformed G3 patients were younger, had lower proliferation indices and were more often treated with SSA; their higher crude event proportions were driven by a shorter observation period rather than by a higher event rate over a comparable time. Once the time-varying Ki-67 > 55% indicator was included in the model, transformed G3 was associated with worse overall survival (HR 1.60, *p* = 0.040). The most plausible reading is that the favorable clinical impression in this group is carried by the lower proliferation index rather than by transformation itself, and that transformation after a period of lower-grade disease may mark an unfavorable trajectory once proliferation is accounted for. This observation rests on 55 patients and 29 deaths and is presented as hypothesis-generating rather than as a firm conclusion; it requires confirmation in independent cohorts. Several mechanisms may contribute to this finding. First, progression from G1/G2 to G3 may select for clones with acquired TP53 or RB1 alterations, which are associated with aggressive biology [9,10]. Second, patients with transformed G3 have usually received several lines of treatment during the lower-grade phase of their disease, which leaves fewer effective options once G3 is documented. Third, pancreatic primaries were more frequent in the transformed group (49.1% vs. 21.3%); in a recent population-based GEP-NEN cohort, pancreatic primaries were associated with a higher hazard of death than small intestinal primaries, although tumor grade was not available in that analysis [31]. Fourth, loss of somatostatin receptor expression during progression, which could not be assessed systematically in the registry, may reduce the benefit of SSA in this subgroup.

### 4.2. Role of Somatostatin Analogues in G3 Neuroendocrine Neoplasms

The use of somatostatin analogues in G3 NEN remains controversial. Current ENETS guidance recommends SSAs primarily for functioning NET G3 with relatively low Ki-67 indices but does not support their use as antiproliferative monotherapy in non-functioning G3 disease [15]. In our cohort, SSA treatment was more common in patients with transformed than in those with de novo G3 tumors (49.1% vs. 9.7%), likely reflecting clinician preference based on prior responsiveness to SSAs in the lower-grade setting or the retention of favorable biological features. However, our multivariable analyses failed to demonstrate a significant association between SSA treatment and improved survival outcomes. This finding challenges the empirical use of SSAs in G3 NEN, even in the potentially more favorable subgroup of transformed G3 tumors. The heterogeneity in somatostatin receptor expression within G3 NEN may influence treatment efficacy. While well-differentiated NET G3 often maintains SSTR2 expression like G1/G2 tumors, poorly differentiated NEC typically demonstrates reduced or absent expression [32]. Therapeutic strategies in grade 3 gastroenteropancreatic NET have recently been reviewed systematically [33]. Several additional factors may contribute to this observation:

First, the apparent worse outcomes in SSA-treated patients in univariable analysis likely reflect selection bias, as patients with more advanced disease or those who progressed on other therapies were more frequently offered SSA treatment. The elimination of this association after multivariable adjustment suggests that the underlying disease biology, rather than SSA treatment itself, primarily determines outcomes. The point estimates for SSA were above 1.0 in every model but reached significance in none. Given that SSA-treated patients differed systematically from untreated patients in age, primary site, functional status, tumor size and proliferation index, these estimates are best read as residual confounding by indication and not as evidence of a harmful effect.

Second, the heterogeneity in somatostatin receptor expression within G3 NEN may influence treatment efficacy, as mentioned above.

Third, the timing of SSA initiation varied considerably within our cohort, with some patients receiving SSAs before G3 diagnosis (primarily in the transformed group) and others after progression on multiple prior therapies. This variability in treatment sequencing may obscure potential benefits in specific clinical scenarios, such as early intervention in transformed G3 tumors with preserved SSTR expression. Exclusion of patients with SSA treatment before the first G3 diagnosis did not have an impact on survival outcomes, although the limited sample size precludes firm conclusions.

Importantly, our findings do not preclude the possibility that certain subsets of G3 patients might benefit from SSA therapy. The absence of a significant detrimental effect on survival (HR 1.32 for PFS and 0.91 for OS, both non-significant) suggests that SSAs may still represent a reasonable option in selected patients, particularly those with functional symptoms requiring control or those with limited alternative options. Conversely, the absence of a statistically significant association does not demonstrate the absence of benefit: in an observational analysis of this size, a modest effect in a subgroup cannot be excluded.

In exploratory subgroup analyses, SSA use was associated with worse PFS in patients without a documented Ki-67 > 55% at any time before baseline (HR 2.12; interaction *p* = 0.010), and two interaction tests for OS were nominally significant without a significant association in any individual subgroup. Given six interaction tests, the small number of SSA-treated patients per subgroup and the likelihood that SSA was preferentially given to patients with progressive disease of lower proliferation, these findings are hypothesis-generating and do not support subgroup-specific recommendations.

With punctual therapies modeled as time-varying cumulative counts, a higher number of surgical procedures was associated with better PFS and OS, and molecular therapy with better PFS. These associations should not be read as treatment effects: surgery is offered to patients with resectable disease and adequate performance status, so that the number of procedures largely reflects patient selection, and the estimate for molecular therapy is imprecise. The same caution applies to surgery of the primary tumor in high-grade disease, where patient selection accounts for much of the apparent benefit [34], and to PRRT, whose place in high-grade GEP-NEN is still being defined [19]. The association of chemotherapy with better overall survival (HR 0.47, *p* = 0.005), which was consistent across primary site, transformation status and Ki-67 category, should be read with the same caution, since treatment allocation in a registry follows performance status and disease extent rather than randomization. Patients fit enough to receive chemotherapy are likely to have a better prognosis irrespective of the treatment. In addition, performance status, tumor burden and disease kinetics are incompletely captured in the registry. This association should therefore not be interpreted as clear evidence of a chemotherapy benefit in this cohort.

### 4.3. Strengths and Limitations

The strengths of our study include its large sample of patients with G3 GEP-NEN, the comprehensive collection of clinicopathological data, and the robust statistical methodology incorporating time-varying treatment effects. The restriction to gastroenteropancreatic primaries aligns the cohort with the classification framework in which the NET G3 category is defined, and the pre-specified, time-varying Ki-67 threshold provides a criterion that can be applied consistently to data spanning several classification eras. However, several limitations must be acknowledged. First, the Ki-67 threshold of 55% is a surrogate for the NET G3/NEC distinction and cannot capture cases in which morphology and proliferation index are discordant, whereas consensus morphological criteria would be the appropriate reference standard [5,7]; in addition, no classifiable Ki-67 value was available for 109 patients (21.0%), who were excluded from the stratified analyses. No multiple imputation was performed, because the indicator is time-varying and its history cannot be reconstructed reliably from registry data; the models without the Ki-67 indicator, which do not require Ki-67 information, yielded the same conclusion for SSA use (Supplementary Tables S9 and S10). In the consensus morphological re-evaluation of 196 high-grade GEP-NEN, Ki-67 overlapped between NET G3 and NEC from 21% to 70%; only two NET G3 exceeded 55%, whereas 29.6% of all cases were NEC with a Ki-67 below 55% [7]. A threshold of 55% therefore identifies NET G3 with high specificity but misclassifies a substantial proportion of NEC with lower proliferation as NET G3-like. Such misclassification would be expected to attenuate rather than create differences between the two Ki-67 categories, so that the hazard ratios for Ki-67 > 55% (PFS 1.40; OS 1.65) are more likely to underestimate than to overestimate the prognostic difference between NEC and NET G3. For SSA use and transformed G3, the Ki-67 indicator acts as an imperfect adjustment variable, and residual confounding by morphology cannot be excluded. Second, the retrospective nature of our analysis introduces potential selection bias and confounding by indication. Performance status, tumor burden and somatostatin receptor expression were not available for adjustment, so that residual confounding remains possible despite time-varying modeling and multivariable adjustment. Third, the lack of centralized pathological review and standardized assessment of somatostatin receptor expression limits our ability to correlate treatment outcomes with tumor biology. Somatostatin receptor imaging (e.g., [68Ga]Ga-DOTATATE PET/CT) was not systematically recorded. As somatostatin receptor expression is a prerequisite for an antiproliferative effect of SSA and frequently decreases with dedifferentiation, the SSA-treated group likely included patients with low or absent receptor expression, which may have diluted a benefit in receptor-positive tumors. Fourth, the heterogeneity in treatment patterns and sequencing complicates the interpretation of SSA efficacy. Fifth, staging information was documented for only 45.5% of patients, which limits the metastatic subgroup analysis, and the transformed G3 group comprises 55 patients, so that all estimates for this group are imprecise. The PRRT covariate is also imprecise, because radionuclide and external beam treatments are recorded under a single registry item and cannot be separated at patient level; the estimate for this covariate can therefore not be attributed to either modality and should be regarded as an adjustment term rather than an estimate of the effectiveness of PRRT. Additionally, our analysis did not include molecular profiling or genetic alterations that might further refine the biological classification of G3 NEN. Finally, we cannot exclude that some de novo G3 NEN, particularly non-functioning tumors, had passed through a lower-grade phase that was not documented in clinical practice. Similarly, we cannot exclude that some G2 NEN that later transformed to G3 already had G3 features in individual metastases. However, the distinct characteristics and differences between the two groups speak against this.

### 4.4. Clinical Implications and Future Directions

Despite these limitations, our findings have several important implications. First, a documented Ki-67 >55% identifies a prognostically distinct subgroup and can be applied to registry data in which a formal NET G3/NEC distinction is not available. Second, the limited impact of SSA therapy on survival outcomes in our multivariable analysis suggests that SSAs should not be routinely recommended for G3 NEN based solely on prior G1/G2 status. However, their use may still be justified in specific clinical scenarios, such as functional symptom control or in tumors with high somatostatin receptor expression. Within the limits of an observational analysis, we suggest the following considerations: (1) SSA therapy in G3 GEP-NEN should primarily be used for symptom control in functioning tumors; (2) in non-functioning tumors, our data do not support continuation of SSA as antiproliferative monotherapy after progression to G3, in line with current ENETS guidance [15]; (3) patients with a Ki-67 > 55% are unlikely to benefit from SSA monotherapy and should be considered for more intensive systemic treatment; (4) transformed G3 tumors, despite their lower Ki-67, did not show a more favorable adjusted survival in this cohort and warrant the same therapeutic consideration as de novo G3. Third, the worse adjusted overall survival of transformed G3 tumors should be examined in cohorts with centralized pathology review before it is allowed to influence clinical decisions. Finally, our study highlights the need for prospective, biomarker-driven trials specifically designed for the heterogeneous population of G3 neuroendocrine neoplasms.

## 5. Conclusions

In this registry-based analysis of 519 patients with G3 gastroenteropancreatic neuroendocrine neoplasms, somatostatin analogue therapy was not associated with improved progression-free or overall survival in any model, and the point estimates are best explained by confounding by indication. Because of the observational design, these estimates reflect observational associations and do not establish a lack of biological efficacy of SSA; residual confounding by unmeasured factors, including performance status, tumor burden and somatostatin receptor status, cannot be excluded. A documented Ki-67 > 55% identified a prognostically distinct subgroup and provides a pragmatic surrogate for the NET G3/NEC distinction in data spanning several classification eras. After adjustment for Ki-67, transformed G3 was associated with worse overall survival, which argues against the assumption that these tumors retain a more favorable biology; given the size of this subgroup, the finding requires confirmation. SSA treatment decisions in G3 disease should be based on functional symptoms, somatostatin receptor status and the individual clinical situation rather than on prior G1/G2 history. Prospective, biomarker-driven studies are needed to further elucidate the role of SSAs and other therapeutic modalities in this heterogeneous population.

## Figures and Tables

**Table 1:** Patient characteristics at baseline, by transformation status. Percentages refer to the total group size; missing values are shown as a separate category and were not included in the statistical tests. Categorical variables were compared with Fisher’s exact test, continuous variables with the Mann–Whitney–Wilcoxon test. CUP, cancer of unknown primary; IQR, interquartile range; SSA, somatostatin analogue.

	De Novo G3 (*N* = 464)	Transformed G3 (*N* = 55)	Total (*N* = 519)	*p*
Sex—*n* (%)				0.083
Female	186 (40.1)	29 (52.7)	215 (41.4)	
Male	276 (59.5)	26 (47.3)	302 (58.2)	
Missing	2 (0.4)	0 (0.0)	2 (0.4)	
Age at diagnosis, years				
Median (IQR)	69.3 (59.0–79.4)	61.1 (52.0–68.6)	68.2 (57.5–78.7)	<0.001
Missing	1 (0.2)	0 (0.0)	1 (0.2)	
Primary site—*n* (%)				<0.001
Pancreas	99 (21.3)	27 (49.1)	126 (24.3)	
Ileum/jejunum	18 (3.9)	7 (12.7)	25 (4.8)	
CUP	77 (16.6)	9 (16.4)	86 (16.6)	
Other	270 (58.2)	12 (21.8)	282 (54.3)	
Functioning tumor—*n* (%)				0.033
No	361 (77.8)	44 (80.0)	405 (78.0)	
Yes	19 (4.1)	6 (10.9)	25 (4.8)	
Not known	84 (18.1)	5 (9.1)	89 (17.1)	
Tumor size at baseline, mm				
Median (IQR)	22.0 (12.0–45.0)	19.5 (15.5–38.5)	20.0 (12.0–45.0)	0.975
Missing	321 (69.2)	39 (70.9)	360 (69.4)	
Ki-67 at baseline, %				
Median (IQR)	70.0 (40.0–90.0)	34.5 (26.5–50.0)	70.0 (35.0–90.0)	<0.001
Missing	114 (24.6)	7 (12.7)	121 (23.3)	
Ki-67 > 55% ever documented—*n* (%)				<0.001
No	116 (25.0)	38 (69.1)	154 (29.7)	
Yes	244 (52.6)	12 (21.8)	256 (49.3)	
Missing	104 (22.4)	5 (9.1)	109 (21.0)	
Ever used SSA—*n* (%)				<0.001
No	419 (90.3)	28 (50.9)	447 (86.1)	
Yes	45 (9.7)	27 (49.1)	72 (13.9)	

**Table 2:** Patient characteristics at baseline, by use of somatostatin analogues. Percentages refer to the total group size; missing values are shown as a separate category and were not included in the statistical tests. CUP, cancer of unknown primary; IQR, interquartile range; SSA, somatostatin analogue.

	No SSA (*N* = 447)	Ever Used SSA (*N* = 72)	Total (*N* = 519)	*p*
Sex—*n* (%)				0.699
Female	187 (41.8)	28 (38.9)	215 (41.4)	
Male	258 (57.7)	44 (61.1)	302 (58.2)	
Missing	2 (0.4)	0 (0.0)	2 (0.4)	
Age at diagnosis, years				
Median (IQR)	69.8 (59.9–79.4)	59.5 (50.0–67.4)	68.2 (57.5–78.7)	<0.001
Missing	1 (0.2)	0 (0.0)	1 (0.2)	
Primary site—*n* (%)				<0.001
Pancreas	86 (19.2)	40 (55.6)	126 (24.3)	
Ileum/jejunum	21 (4.7)	4 (5.6)	25 (4.8)	
CUP	72 (16.1)	14 (19.4)	86 (16.6)	
Other	268 (60.0)	14 (19.4)	282 (54.3)	
Functioning tumor—*n* (%)				<0.001
No	351 (78.5)	54 (75.0)	405 (78.0)	
Yes	14 (3.1)	11 (15.3)	25 (4.8)	
Not known	82 (18.3)	7 (9.7)	89 (17.1)	
Tumor size at baseline, mm				
Median (IQR)	20.0 (11.5–45.0)	40.0 (20.0–75.0)	20.0 (12.0–45.0)	0.014
Missing	305 (68.2)	55 (76.4)	360 (69.4)	
Ki-67 at baseline, %				
Median (IQR)	70.0 (45.0–90.0)	30.0 (25.0–56.0)	70.0 (35.0–90.0)	<0.001
Missing	108 (24.2)	13 (18.1)	121 (23.3)	
G1 or G2 grading before G3—*n* (%)				<0.001
No	419 (93.7)	45 (62.5)	464 (89.4)	
Yes	28 (6.3)	27 (37.5)	55 (10.6)	
Ki-67 > 55% ever documented—*n* (%)				<0.001
No	108 (24.2)	46 (63.9)	154 (29.7)	
Yes	240 (53.7)	16 (22.2)	256 (49.3)	
Missing	99 (22.1)	10 (13.9)	109 (21.0)	

**Table 3:** Multivariable Cox proportional hazards models for progression-free survival and overall survival, including the Ki-67 > 55% indicator (*n* = 410). Sex and transformed G3 status are fixed; use of SSA, chemotherapy and molecular therapy, the cumulative numbers of punctual therapies and the Ki-67 > 55% indicator are time-varying.

Covariate	PFS—HR (95% CI)	PFS—*p*	OS—HR (95% CI)	OS—*p*
Use of SSA (y/*n*)	1.32 (0.85–2.04)	0.215	0.91 (0.54–1.56)	0.744
Ki-67 > 55% (y/*n*)	1.40 (1.06–1.84)	0.016	1.65 (1.15–2.37)	0.006
Transformed G3 (y/*n*)	1.11 (0.75–1.63)	0.609	1.60 (1.02–2.51)	0.040
Sex (female vs. male)	1.19 (0.91–1.55)	0.201	1.14 (0.81–1.60)	0.447
Age	1.01 (1.00–1.02)	0.089	1.02 (1.00–1.03)	0.010
Number of surgeries (0–2)	0.58 (0.46–0.74)	<0.001	0.48 (0.35–0.66)	<0.001
Chemotherapy (y/*n*)	0.74 (0.52–1.05)	0.093	0.47 (0.28–0.79)	0.005
Molecular therapy (y/*n*)	0.32 (0.12–0.89)	0.028	1.03 (0.50–2.11)	0.945
Number of ablative therapies (0–1)	1.33 (0.61–2.90)	0.472	1.40 (0.69–2.85)	0.351
Number of PRRT and other irradiation treatments (0–4)	1.01 (0.85–1.20)	0.913	1.11 (0.94–1.30)	0.212

HR, hazard ratio; CI, confidence interval; PFS, progression-free survival; OS, overall survival; SSA, somatostatin analogue. Models were fitted in Stata 19.0. The PRRT covariate derives from a single registry item covering radionuclide and external beam treatments; PRRT accounts for approximately 70–75% of these. Counts of punctual therapies were capped at 2 (surgery), 1 (ablative therapy) and 4 (PRRT and other irradiation) and entered as ordinal terms; hazard ratios refer to each additional treatment.

## Data Availability

Restrictions apply to the availability of these data. The data were obtained from SwissNET and are available from the corresponding author with the permission of the SwissNET steering committee.

## Supplementary Materials

The following supporting information can be downloaded at: https://www.mdpi.com/article/10.3390/cancers18203357/s1, Table S1: Comparison of patients with and without classifiable Ki-67; Table S2: Number of punctual therapies per patient; Table S3: Follow-up, treatment sequence and liver metastases among SSA-treated patients; Table S4: SSA substances, formulations and documented dosages; Table S5: Patient characteristics at baseline by Ki-67 stratification; Table S6: Occurrence of outcomes by transformation status; Table S7: Occurrence of outcomes by SSA treatment status; Table S8: Occurrence of outcomes by staging; Table S9: Cox models for progression-free survival, full cohort and excluding patients who started SSA before baseline; Table S10: Cox models for overall survival, full cohort and excluding patients who started SSA before baseline; Table S11: Subgroup analyses of the association between chemotherapy and overall survival; Table S12: Subgroup analyses of the association between SSA use and survival; Figure S1: Flow of patients through the study; Figure S2: Forest plot of subgroup-specific hazard ratios for chemotherapy (overall survival); Figure S3: Kaplan–Meier curves for progression-free survival, excluding patients who started SSA before baseline; Figure S4: Kaplan–Meier curves for overall survival, excluding patients who started SSA before baseline; Figure S5: Forest plots of subgroup-specific hazard ratios for SSA use (progression-free and overall survival).

## References

[1] Klöppel G. (2017). Neuroendocrine Neoplasms: Dichotomy, Origin and Classifications. Visc. Med..

[2] WHO Classification of Tumours Editorial Board (2019). Digestive System Tumours.

[3] Rindi G., Klimstra D.S., Abedi-Ardekani B., Asa S.L., Bosman F.T., Brambilla E., Busam K.J., de Krijger R.R., Dietel M., El-Naggar A.K. (2018). A Common Classification Framework for Neuroendocrine Neoplasms: An International Agency for Research on Cancer (IARC) and World Health Organization (WHO) Expert Consensus Proposal. Mod. Pathol..

[4] Nagtegaal I.D., Odze R.D., Klimstra D., Paradis V., Rugge M., Schirmacher P., Washington K.M., Carneiro F., Cree I.A. (2020). WHO Classification of Tumours Editorial Board. The 2019 WHO Classification of Tumours of the Digestive System. Histopathology.

[5] Rindi G., Mete O., Uccella S., Basturk O., La Rosa S., Brosens L.A.A., Ezzat S., de Herder W.W., Klimstra D.S., Papotti M. (2022). Overview of the 2022 WHO Classification of Neuroendocrine Neoplasms. Endocr. Pathol..

[6] Sorbye H., Baudin E., Perren A. (2018). The Problem of High-Grade Gastroenteropancreatic Neuroendocrine Neoplasms: Well-Differentiated Neuroendocrine Tumors, Neuroendocrine Carcinomas, and Beyond. Endocrinol. Metab. Clin. N. Am..

[7] Elvebakken H., Perren A., Scoazec J.-Y., Tang L.H., Federspiel B., Klimstra D.S., Vestermark L.W., Ali A.S., Zlobec I., Myklebust T.Å. (2021). A Consensus-Developed Morphological Re-Evaluation of 196 High-Grade Gastroenteropancreatic Neuroendocrine Neoplasms and Its Clinical Correlations. Neuroendocrinology.

[8] Velayoudom-Cephise F.L., Duvillard P., Foucan L., Hadoux J., Chougnet C.N., Leboulleux S., Malka D., Guigay J., Goere D., Debaere T. (2013). Are G3 ENETS Neuroendocrine Neoplasms Heterogeneous?. Endocr. Relat. Cancer.

[9] Joseph N.M., Umetsu S.E., Kim G.E., Gai W., Gonda R.M., Komashko V.M., Barkovich K.J., Cho S.J., Meyerson M. (2024). Progression of Low-Grade Neuroendocrine Tumors (NET) to High-Grade Neoplasms Harboring the NEC-like Co-Alteration of RB1 and TP53. Endocr. Pathol..

[10] Venizelos A., Elvebakken H., Perren A., Nikolaienko O., Deng W., Lothe I.M.B., Couvelard A., Hjortland G.O., Sundlöv A., Svensson J. (2021). The Molecular Characteristics of High-Grade Gastroenteropancreatic Neuroendocrine Neoplasms. Endocr. Relat. Cancer.

[11] Tang L.H., Basturk O., Sue J.J., Klimstra D.S. (2016). A Practical Approach to the Classification of WHO Grade 3 (G3) Well-Differentiated Neuroendocrine Tumor (WD-NET) and Poorly Differentiated Neuroendocrine Carcinoma (PD-NEC) of the Pancreas. Am. J. Surg. Pathol..

[12] Rinke A., Müller H.H., Schade-Brittinger C., Klose K.J., Barth P., Wied M., Mayer C., Aminossadati B., Pape U.F., Bläker M. (2009). Placebo-Controlled, Double-Blind, Prospective, Randomized Study on the Effect of Octreotide LAR in the Control of Tumor Growth in Patients with Metastatic Neuroendocrine Midgut Tumors: A Report from the PROMID Study Group. J. Clin. Oncol..

[13] Caplin M.E., Pavel M., Ćwikła J.B., Phan A.T., Raderer M., Sedláčková E., Cadiot G., Wolin E.M., Capdevila J., Wall L. (2014). Lanreotide in Metastatic Enteropancreatic Neuroendocrine Tumors. N. Engl. J. Med..

[14] Pavel M., Ćwikła J.B., Lombard-Bohas C., Borbath I., Shah T., Pape U.F., Capdevila J., Panzuto F., Truong Thanh X.M., Houchard A. (2021). Efficacy and Safety of High-Dose Lanreotide Autogel in Patients with Progressive Pancreatic or Midgut Neuroendocrine Tumours: CLARINET FORTE Phase 2 Study Results. Eur. J. Cancer.

[15] McNamara M.G., Sorbye H., Begum N., Christ E., Fazio N., Fernandez-Cuesta L., Garcia-Carbonero R., Kaltsas G., Kasajima A., Salazar R. (2025). Controversies in NEN: An ENETS Position Statement on the Treatment of Patients with Grade 3 Well-Differentiated Neuroendocrine Tumours of the Gastro-Enteropancreatic Tract. J. Neuroendocrinol..

[16] Singh S., Halperin D., Myrehaug S., Herrmann K., Pavel M., Gao B., Grozinsky-Glasberg S., Wolin E.M., Gorbunova V., Kulke M.H. (2024). [177Lu]Lu-DOTA-TATE Plus Long-Acting Octreotide versus High-Dose Long-Acting Octreotide for the Treatment of Newly Diagnosed, Advanced Grade 2–3, Well-Differentiated, Gastroenteropancreatic Neuroendocrine Tumours (NETTER-2): An Open-Label, Randomised, Phase 3 Study. Lancet.

[17] Singh S., Halperin D., Myrehaug S., Herrmann K., Pavel M., Gao B., Grozinsky-Glasberg S., Wolin E.M., Gorbunova V., Kulke M.H. (2024). First-Line Efficacy of [177Lu]Lu-DOTA-TATE in Patients with Advanced Grade 2 and Grade 3, Well-Differentiated Gastroenteropancreatic Neuroendocrine Tumors by Tumor Grade and Primary Origin: Subgroup Analysis of the Phase III NETTER-2 Study. Ann. Oncol..

[18] Garcia-Carbonero R., Sorbye H., Baudin E., Raymond E., Wiedenmann B., Niederle B., Sedlackova E., Toumpanakis C., Anlauf M., Cwikla J.B. (2016). ENETS Consensus Guidelines for High-Grade Gastroenteropancreatic Neuroendocrine Tumors and Neuroendocrine Carcinomas. Neuroendocrinology.

[19] Sorbye H., Kong G., Grozinsky-Glasberg S. (2020). PRRT in High-Grade Gastroenteropancreatic Neuroendocrine Neoplasms (WHO G3). Endocr. Relat. Cancer.

[20] Sorbye H., Hjortland G.O., Vestermark L.W., Ladekarl M., Svensson J., Sundlöv A., Janson E.T., Garresori H., Hofsli E., Kersten C. (2025). Characteristics and Treatment Outcome in a Prospective Cohort of 639 Advanced High-Grade Digestive Neuroendocrine Neoplasms (NET G3 and NEC). The NORDIC NEC 2 Study. Br. J. Cancer.

[21] Heetfeld M., Chougnet C.N., Olsen I.H., Rinke A., Borbath I., Crespo G., Barriuso J., Pavel M., O’Toole D., Walter T. (2015). Characteristics and Treatment of Patients with G3 Gastroenteropancreatic Neuroendocrine Neoplasms. Endocr. Relat. Cancer.

[22] Sorbye H., Welin S., Langer S.W., Vestermark L.W., Holt N., Osterlund P., Dueland S., Hofsli E., Guren M.G., Ohrling K. (2013). Predictive and Prognostic Factors for Treatment and Survival in 305 Patients with Advanced Gastrointestinal Neuroendocrine Carcinoma (WHO G3): The NORDIC NEC Study. Ann. Oncol..

[23] Suissa S. (2008). Immortal Time Bias in Pharmacoepidemiology. Am. J. Epidemiol..

[24] Lévesque L.E., Hanley J.A., Kezouh A., Suissa S. (2010). Problem of Immortal Time Bias in Cohort Studies: Example Using Statins for Preventing Progression of Diabetes. BMJ.

[25] Hijioka S., Hosoda W., Mizuno N., Hara K., Imaoka H., Bhatia V., Mekky M.A., Uno N., Ogura T., Tajika M. (2015). Does the WHO 2010 Classification of Pancreatic Neuroendocrine Neoplasms Accurately Characterize Pancreatic Neuroendocrine Carcinomas?. J. Gastroenterol..

[26] Basturk O., Yang Z., Tang L.H., Hruban R.H., Adsay V., McCall C.M., Krasinskas A.M., Jang K.T., Frankel W.L., Balci S. (2015). The High-Grade (WHO G3) Pancreatic Neuroendocrine Tumor Category Is Morphologically and Biologically Heterogeneous and Includes Both Well Differentiated and Poorly Differentiated Neoplasms. Am. J. Surg. Pathol..

[27] Raj N., Valentino E., Capanu M., Tang L.H., Basturk O., Untch B.R., Allen P.J., Klimstra D.S., Reidy-Lagunes D. (2017). Treatment Response and Outcomes of Grade 3 Pancreatic Neuroendocrine Neoplasms Based on Morphology: Well Differentiated versus Poorly Differentiated. Pancreas.

[28] Coriat R., Walter T., Terris B., Couvelard A., Ruszniewski P. (2016). Gastroenteropancreatic Well-Differentiated Grade 3 Neuroendocrine Tumors: Review and Position Statement. Oncologist.

[29] Konukiewitz B., Schlitter A.M., Jesinghaus M., Pfister D., Steiger K., Segler A., Agaimy A., Sipos B., Zamboni G., Weichert W. (2017). Somatostatin Receptor Expression Related to TP53 and RB1 Alterations in Pancreatic and Extrapancreatic Neuroendocrine Neoplasms with a Ki67-Index above 20%. Mod. Pathol..

[30] Xue Y., Reid M.D., Pehlivanoglu B., Obeng R.C., Jiang H., Memis B., Lui S.K., Sarmiento J., Kooby D., Maithel S.K. (2020). Morphologic Variants of Pancreatic Neuroendocrine Tumors: Clinicopathologic Analysis and Prognostic Stratification. Endocr. Pathol..

[31] Hallet J., Meloche-Dumas L., Sarzo C., Court C., Armah J., Ding A., Chan W.C., Singh S. (2026). Incidence and Survival of Gastro-Entero-Pancreatic Neuroendocrine Neoplasms in the Contemporary Era. JAMA Netw. Open.

[32] Hofland J., Kaltsas G., de Herder W.W. (2020). Advances in the Diagnosis and Management of Well-Differentiated Neuroendocrine Neoplasms. Endocr. Rev..

[33] Donadio M.D., Brito A.B., Riechelmann R.P. (2023). A Systematic Review of Therapeutic Strategies in Gastroenteropancreatic Grade 3 Neuroendocrine Tumors. Ther. Adv. Med. Oncol..

[34] Pommergaard H.C., Nielsen K., Sorbye H., Federspiel B., Tabaksblat E.M., Vestermark L.W., Hansen C.P., Falkmer U.G., Knigge U. (2021). Surgery of the Primary Tumour in 201 Patients with High-Grade Gastroenteropancreatic Neuroendocrine and Mixed Neuroendocrine–Non-Neuroendocrine Neoplasms. J. Neuroendocrinol..

