# Supplementary material for: Somatostatin Analogues Do Not Improve Survival in Grade 3 Gastroenteropancreatic Neuroendocrine Neoplasms: A Registry-Based Analysis with Ki-67 Stratification
Source: Cancers (Basel). 2026 Oct 17;18(20):3357. doi: 10.3390/cancers18203357 (PMC13702314; doi:10.3390/cancers18203357)
Supplement: Supplementary file 1 [file cancers-18-03357-s001.zip › cancers-4593557-supplementary.pdf]

Supplementary Materials

Somatostatin Analogues Do Not Improve Survival in Grade 3 Gastroenteropancreatic Neuroendocrine Neoplasms: A Registry-Based Analysis with Ki-67 Stratification

Alexander R. Siebenhüner, Jean-Benoît Rossel, Emanuel Christ and Attila Kollár

Contents

Table S1. Comparison of patients with and without a classifiable Ki-67 value.

Table S2. Number of punctual therapies per patient after baseline.

Table S3. Follow-up, treatment sequence and liver metastases among patients who ever used somatostatin analogues (N = 72), by transformation status.

Table S4. SSA substances, formulations and documented dosages.

Table S5. Patient characteristics at baseline, by Ki-67 stratification (Ki-67 analysis population, n = 410).

Table S6. Occurrence of outcomes, by transformation status.

Table S7. Occurrence of outcomes, by use of somatostatin analogues.

Table S8. Occurrence of outcomes by staging, among the 236 patients with documented TNM staging.

Table S9. Cox proportional hazards models for progression-free survival: full cohort, and after exclusion of patients who started SSA before baseline.

Table S10. Cox proportional hazards models for overall survival: full cohort, and after exclusion of patients who started SSA before baseline.

Table S11. Subgroup analyses of the association between chemotherapy and overall survival.

Table S12. Subgroup analyses of the association between SSA use and progression-free and overall survival.

Figure S1. Flow of patients through the study.

Figure S2. Forest plot of subgroup-specific hazard ratios for chemotherapy and overall survival.

Figure S3. Kaplan-Meier curves for progression-free survival, excluding patients who started SSA before baseline.

Figure S4. Kaplan-Meier curves for overall survival, excluding patients who started SSA before baseline.

Figure S5. Forest plots of subgroup-specific hazard ratios for SSA use.

Table S1. Comparison of patients with and without a classifiable Ki-67 value. Percentages refer to the total group size; missing values are shown as a separate category and were not included in the statistical tests.

	Available (N = 410)	Missing (N = 109)	Total (N = 519)	p
Sex — n (%)				0.743
Female	168 (41.0)	47 (43.1)	215 (41.4)	
Male	240 (58.5)	62 (56.9)	302 (58.2)	
Missing	2 (0.5)	0 (0.0)	2 (0.4)	
Age at diagnosis, years				
Median (IQR)	67.5 (56.1–77.7)	71.8 (62.0–80.2)	68.2 (57.5–78.7)	0.015
Missing	1 (0.2)	0 (0.0)	1 (0.2)	
Primary site — n (%)				0.002
Pancreas	113 (27.6)	13 (11.9)	126 (24.3)	
Ileum/jejunum	22 (5.4)	3 (2.8)	25 (4.8)	
CUP	65 (15.9)	21 (19.3)	86 (16.6)	
Other	210 (51.2)	72 (66.1)	282 (54.3)	
Functioning tumor — n (%)				0.399
No	321 (78.3)	84 (77.1)	405 (78.0)	
Yes	22 (5.4)	3 (2.8)	25 (4.8)	
Not known	67 (16.3)	22 (20.2)	89 (17.1)	
Tumor size at baseline, mm				
Median (IQR)	20.0 (12.0–45.0)	22.0 (12.0–40.0)	20.0 (12.0–45.0)	0.716
Missing	280 (68.3)	80 (73.4)	360 (69.4)	
G1 or G2 grading before G3 — n (%)				0.022
No	360 (87.8)	104 (95.4)	464 (89.4)	
Yes	50 (12.2)	5 (4.6)	55 (10.6)	
Ever used SSA — n (%)				0.121
No	348 (84.9)	99 (90.8)	447 (86.1)	
Yes	62 (15.1)	10 (9.2)	72 (13.9)	
Staging — n (%)				0.862
Localized	108 (26.3)	21 (19.3)	129 (24.9)	
Metastatic	88 (21.5)	19 (17.4)	107 (20.6)	
Missing	214 (52.2)	69 (63.3)	283 (54.5)	

Categorical variables were compared with Fisher's exact test and continuous variables with the Mann-Whitney-Wilcoxon test. CUP, cancer of unknown primary; IQR, interquartile range; SSA, somatostatin analogue.

Table S2. Number of punctual therapies per patient after baseline (N = 519).

Number of treatments	n (%)
Surgery	
0	277 (53.4)
1	199 (38.3)
2	34 (6.6)
3	8 (1.5)
4	1 (0.2)
Ablative therapy	
0	488 (94.0)
1	20 (3.9)

Number of treatments	n (%)
2	5 (1.0)
3	5 (1.0)
4	1 (0.2)
PRRT and other irradiation	
0	389 (75.0)
1	50 (9.6)
2	28 (5.4)
3	13 (2.5)
4	19 (3.7)
5	8 (1.5)
6	5 (1.0)
8	4 (0.8)
9	2 (0.4)
10	1 (0.2)

In the Cox models, counts were capped at 2 (surgery), 1 (ablative therapy) and 4 (PRRT and other irradiation) and entered as time-varying ordinal terms. PRRT, peptide receptor radionuclide therapy.

Table S3. Follow-up, treatment sequence and liver metastases among patients who ever used somatostatin analogues (N = 72), by transformation status.

	De novo G3 (N = 45)	Transformed G3 (N = 27)	Total (N = 72)	p
Observation time, years — median (IQR)	3.0 (0.9–5.8)	1.1 (0.5–2.9)	1.8 (0.7–4.6)	0.042
Time on SSA, years — median (IQR)	0.9 (0.4–2.2)	0.5 (0.0–1.2)	0.8 (0.2–1.9)	0.058
First use of SSA before G3 diagnosis — n (%)				<0.001
No	39 (86.7)	10 (37.0)	49 (68.1)	
Yes	6 (13.3)	17 (63.0)	23 (31.9)	
Any therapy before SSA — n (%)				0.028
No	8 (17.8)	12 (44.4)	20 (27.8)	
Yes	37 (82.2)	15 (55.6)	52 (72.2)	
Surgery	25 (67.6)	9 (60.0)	34 (65.4)	0.749
Chemotherapy	20 (54.1)	12 (80.0)	32 (61.5)	0.118
Ablative therapy	3 (8.1)	3 (20.0)	6 (11.5)	0.338
Irradiation	13 (35.1)	7 (46.7)	20 (38.5)	0.534
Any therapy after first use and before last stop of SSA — n (%)				1.000
No	19 (42.2)	12 (44.4)	31 (43.1)	
Yes	26 (57.8)	15 (55.6)	41 (56.9)	
Any therapy after last stop of SSA — n (%)				0.314
No	31 (68.9)	15 (55.6)	46 (63.9)	
Yes	14 (31.1)	12 (44.4)	26 (36.1)	
Ever had liver metastasis — n (%)				0.065
No	12 (26.7)	2 (7.4)	14 (19.4)	
Yes	33 (73.3)	25 (92.6)	58 (80.6)	

Table S4. SSA substances, formulations and documented dosages among patients who ever used somatostatin analogues (N = 72).

	Total (N = 72)
Therapy pathway — n (%)	
Lanreotide (old formulation) LAR (90 mg every 28 days)	1 (1.4)
Lanreotide	33 (45.8)
Lanreotide (120 mg every 28 days)	1 (1.4)
Lanreotide, then octreotide LAR	2 (2.8)
Octreotide LAR	18 (25.0)
Octreotide LAR (10 mg, then 20 mg, then 30 mg every 28 days)	1 (1.4)
Octreotide LAR (20 mg every 28 days)	2 (2.8)
Octreotide LAR (20 mg every 28 days), then octreotide s.c. (200 µg daily), then lanreotide (old formulation) LAR (90 mg, then 120 mg every 28 days)	1 (1.4)
Octreotide LAR (20 mg, then 30 mg every 28 days)	1 (1.4)
Octreotide LAR (30 mg every 28 days)	1 (1.4)
Octreotide LAR (30 mg, then 40 mg every 28 days), then lanreotide	1 (1.4)
Octreotide LAR, then lanreotide	1 (1.4)
Octreotide s.c.	2 (2.8)
Octreotide s.c. (30 mg every 28 days), then octreotide LAR	2 (2.8)
Octreotide s.c., then lanreotide	2 (2.8)
Octreotide s.c., then octreotide LAR	1 (1.4)
Octreotide s.c., then octreotide LAR (20 mg, then 30 mg every 28 days)	1 (1.4)
Pasireotide, then octreotide LAR	1 (1.4)
Ever used — n (%)	
Lanreotide (old formulation) LAR	2 (2.8)
Lanreotide	40 (55.6)
Octreotide s.c.	9 (12.5)
Octreotide LAR	33 (45.8)
Pasireotide	1 (1.4)

Pathways and dosages as documented in the SwissNET registry; dosages were documented for 12 patients (16.7%). The median duration of SSA treatment was 0.8 years (IQR 0.2–1.9; Table S3). LAR, long-acting release; s.c., subcutaneous; SSA, somatostatin analogue.

Table S5. Patient characteristics at baseline, by Ki-67 stratification (Ki-67 analysis population, n = 410). Percentages refer to the total group size; missing values are shown as a separate category and were not included in the statistical tests.

	Never Ki-67 >55% (N = 154)	Ki-67 >55% at least once (N = 256)	Total (N = 410)	p
Sex — n (%)				<0.001
Female	80 (51.9)	88 (34.4)	168 (41.0)	
Male	73 (47.4)	167 (65.2)	240 (58.5)	
Missing	*1 (0.6)*	*1 (0.4)*	*2 (0.5)*	
Age at diagnosis, years				
Median (IQR)	63.3 (54.2–75.1)	69.4 (59.1–80.1)	67.5 (56.1–77.7)	<0.001
Missing	*0 (0.0)*	*1 (0.4)*	*1 (0.2)*	
Primary site — n (%)				<0.001
Pancreas	77 (50.0)	36 (14.1)	113 (27.6)	
Ileum/jejunum	14 (9.1)	8 (3.1)	22 (5.4)	
CUP	21 (13.6)	44 (17.2)	65 (15.9)	
Other	42 (27.3)	168 (65.6)	210 (51.2)	

	**Never Ki-67 >55% (N = 154)**	**Ki-67 >55% at least once (N = 256)**	**Total (N = 410)**	**p**
Functioning tumor — n (%)				0.444
No	117 (76.0)	204 (79.7)	321 (78.3)	
Yes	11 (7.1)	11 (4.3)	22 (5.4)	
Not known	26 (16.9)	41 (16.0)	67 (16.3)	
Tumor size at baseline, mm				
Median (IQR)	23.0 (15.0–45.0)	18.8 (10.5–49.0)	20.0 (12.0–45.0)	0.405
Missing	*100 (64.9)*	*180 (70.3)*	*280 (68.3)*	
Ki-67 at baseline, %				
Median (IQR)	30.0 (25.0–40.0)	80.0 (70.0–90.0)	70.0 (36.0–90.0)	<0.001
Missing	*8 (5.2)*	*5 (2.0)*	*13 (3.2)*	
G1 or G2 grading before G3 — n (%)				<0.001
No	116 (75.3)	244 (95.3)	360 (87.8)	
Yes	38 (24.7)	12 (4.7)	50 (12.2)	
Ever used SSA — n (%)				<0.001
No	108 (70.1)	240 (93.8)	348 (84.9)	
Yes	46 (29.9)	16 (6.2)	62 (15.1)	
Staging — n (%)				0.004
Localized	41 (26.6)	67 (26.2)	108 (26.3)	
Metastatic	52 (33.8)	36 (14.1)	88 (21.5)	
Missing	*61 (39.6)*	*153 (59.8)*	*214 (52.2)*	

Table S6. Occurrence of outcomes, by transformation status.

	De novo G3 (N = 464)	Transformed G3 (N = 55)	Total (N = 519)	p
Progression, relapse or death — n (%)	273 (58.8)	41 (74.5)	314 (60.5)	0.028
Death — n (%)	162 (34.9)	29 (52.7)	191 (36.8)	0.012

Table S7. Occurrence of outcomes, by use of somatostatin analogues.

	No SSA (N = 447)	Ever used SSA (N = 72)	Total (N = 519)	p
Progression, relapse or death — n (%)	250 (55.9)	64 (88.9)	314 (60.5)	<0.001
Death — n (%)	151 (33.8)	40 (55.6)	191 (36.8)	<0.001

Table S8. Occurrence of outcomes by staging, among the 236 patients with documented TNM staging.

	Localized (N = 129)	Metastatic (N = 107)	Total (N = 236)	p
Progression, relapse or death — n (%)	66 (51.2)	89 (83.2)	155 (65.7)	<0.001
Death — n (%)	36 (27.9)	50 (46.7)	86 (36.4)	0.004

Table S9. Cox proportional hazards models for progression-free survival: full cohort, and after exclusion of patients who started SSA before baseline. Models without the Ki-67 indicator. Sex and transformed G3 status are fixed; use of SSA, chemotherapy and molecular therapy and the cumulative numbers of punctual therapies are time-varying.

Covariate	Full cohort (N = 519) HR (95% CI)	p	Excluding pre-baseline SSA (N = 496) HR (95% CI)	p
Use of SSA (y/n)	1.46 (0.98–2.17)	0.060	1.30 (0.82–2.08)	0.267
Transformed G3 (y/n)	1.18 (0.81–1.71)	0.396	1.07 (0.68–1.68)	0.777
Sex (female vs male)	1.06 (0.84–1.34)	0.626	1.06 (0.83–1.35)	0.631
Age	1.00 (1.00–1.01)	0.368	1.00 (1.00–1.01)	0.382
Number of surgeries (0–2)	0.59 (0.48–0.72)	<0.001	0.59 (0.48–0.73)	<0.001
Chemotherapy (y/n)	0.87 (0.63–1.20)	0.396	0.91 (0.66–1.26)	0.561
Molecular therapy (y/n)	0.34 (0.13–0.93)	0.035	0.11 (0.01–0.77)	0.026
Number of ablative therapies (0–1)	1.24 (0.57–2.70)	0.581	1.37 (0.59–3.15)	0.464
Number of PRRT and other irradiation treatments (0–4)	1.05 (0.89–1.24)	0.538	1.07 (0.89–1.27)	0.474

HR, hazard ratio; CI, confidence interval; SSA, somatostatin analogue. The PRRT covariate derives from a single registry item covering radionuclide and external beam treatments; PRRT accounts for approximately 70–75% of these. Counts of punctual therapies were capped at 2 (surgery), 1 (ablative therapy) and 4 (PRRT and other irradiation) and entered as ordinal terms; hazard ratios refer to each additional treatment. PRRT, peptide receptor radionuclide therapy.

Table S10. Cox proportional hazards models for overall survival: full cohort, and after exclusion of patients who started SSA before baseline. Models without the Ki-67 indicator. Sex and transformed G3 status are fixed; use of SSA, chemotherapy and molecular therapy and the cumulative numbers of punctual therapies are time-varying.

Covariate	Full cohort (N = 519) HR (95% CI)	p	Excluding pre-baseline SSA (N = 496) HR (95% CI)	p
Use of SSA (y/n)	0.98 (0.60–1.61)	0.945	0.76 (0.40–1.42)	0.385
Transformed G3 (y/n)	1.51 (0.98–2.32)	0.060	1.10 (0.62–1.93)	0.752
Sex (female vs male)	1.03 (0.76–1.39)	0.868	1.04 (0.75–1.43)	0.819
Age	1.01 (1.00–1.02)	0.008	1.01 (1.00–1.02)	0.015
Number of surgeries (0–2)	0.50 (0.38–0.65)	<0.001	0.53 (0.40–0.71)	<0.001
Chemotherapy (y/n)	0.60 (0.38–0.94)	0.027	0.65 (0.41–1.04)	0.076
Molecular therapy (y/n)	1.04 (0.50–2.14)	0.920	0.78 (0.32–1.94)	0.596
Number of ablative therapies (0–1)	1.36 (0.70–2.64)	0.369	1.29 (0.58–2.84)	0.530
Number of PRRT and other irradiation treatments (0–4)	1.16 (1.00–1.35)	0.043	1.21 (1.04–1.42)	0.014

HR, hazard ratio; CI, confidence interval; SSA, somatostatin analogue. The PRRT covariate derives from a single registry item covering radionuclide and external beam treatments; PRRT accounts for approximately 70–75% of these. Counts of punctual therapies were capped at 2 (surgery), 1 (ablative therapy) and 4 (PRRT and other irradiation) and entered as ordinal terms; hazard ratios refer to each additional treatment. PRRT, peptide receptor radionuclide therapy.

Table S11. Subgroup analyses of the association between chemotherapy (time-varying) and overall survival.

Subgroup	HR (95% CI; p)	p for interaction
Pancreatic primary	0.75 (0.31–1.82; 0.526)	0.202
Non-pancreatic primary	0.37 (0.19–0.71; 0.003)	
Transformed G3	0.65 (0.22–1.88; 0.423)	0.479
De novo G3	0.41 (0.23–0.76; 0.004)	
Ki-67 >55% documented before baseline	0.39 (0.19–0.78; 0.008)	0.352
No Ki-67 >55% documented before baseline	0.64 (0.29–1.43; 0.280)	
Overall	0.47 (0.28–0.79; 0.005)	

Hazard ratios from the main Cox model for overall survival (Table 3 of the main manuscript), with an interaction term between the subgroup variable and chemotherapy. Overall: hazard ratio for chemotherapy in the main model. CI, confidence interval; HR, hazard ratio.

Table S12. Subgroup analyses of the association between SSA use (time-varying) and progression-free and overall survival.

Subgroup	PFS: HR (95% CI; p)	p for interaction	OS: HR (95% CI; p)	p for interaction
Pancreatic primary	1.76 (0.95–3.28; 0.074)	0.221	1.37 (0.66–2.83; 0.398)	0.235
Non-pancreatic primary	1.03 (0.55–1.91; 0.931)		0.71 (0.31–1.63; 0.417)	
Transformed G3	1.80 (0.77–4.20; 0.176)	0.417	1.75 (0.79–3.88; 0.171)	0.049
De novo G3	1.19 (0.72–1.99; 0.499)		0.58 (0.27–1.25; 0.165)	
Ki-67 >55% documented before baseline	0.59 (0.25–1.37; 0.218)	0.010	0.34 (0.10–1.10; 0.073)	0.027
No Ki-67 >55% documented before baseline	2.12 (1.27–3.53; 0.004)		1.49 (0.80–2.76; 0.207)	
Overall	1.32 (0.85–2.04; 0.215)		0.91 (0.54–1.56; 0.744)	

Hazard ratios from the main Cox model including the time-varying Ki-67 indicator (Table 3 of the main manuscript), with an interaction term between the subgroup variable and SSA use. Baseline is the date of the first G3 diagnosis. Overall: hazard ratio for SSA use in the main model. CI, confidence interval; HR, hazard ratio; OS, overall survival; PFS, progression-free survival; SSA, somatostatin analogue.

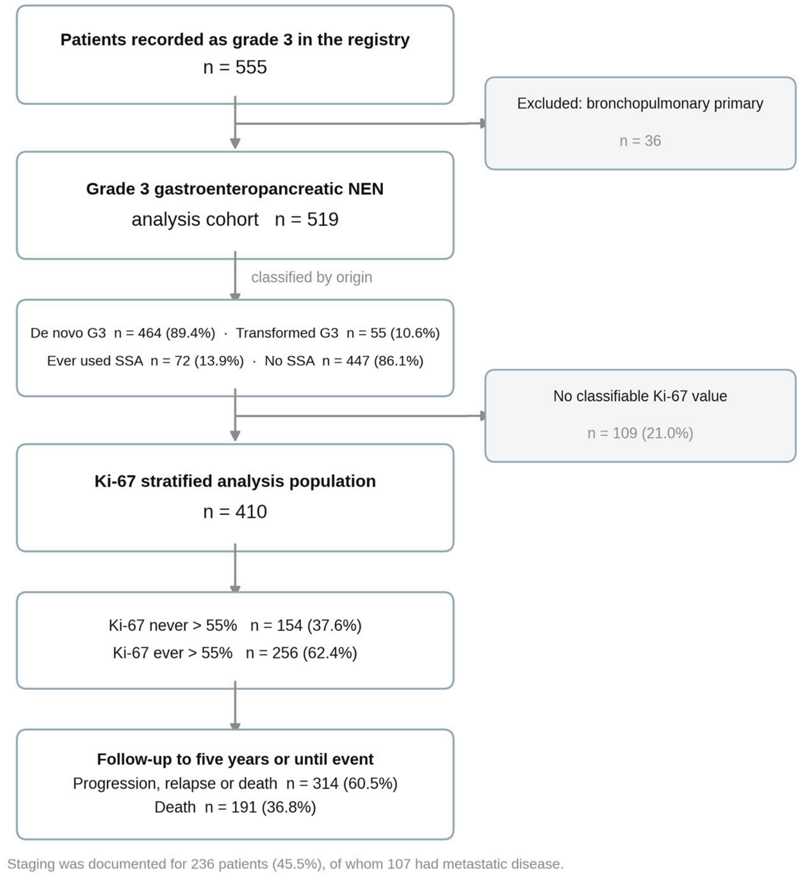

Figure S1. Flow of patients through the study. Patients with a bronchopulmonary primary were excluded before analysis; patients without any classifiable Ki-67 value could not be assigned to a Ki-67 stratum and were excluded from the stratified analyses only.

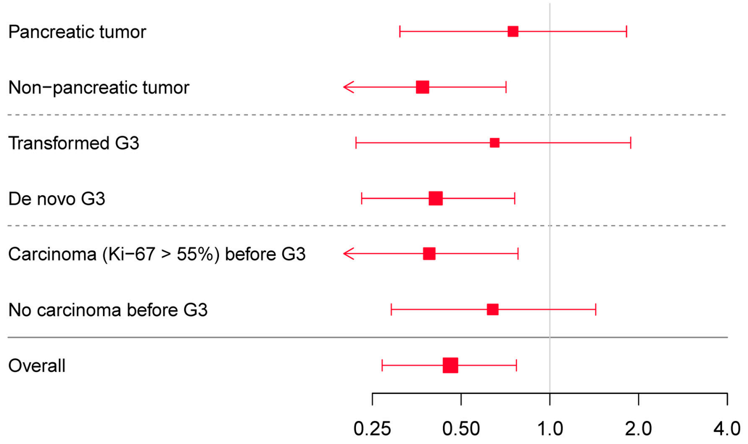

Figure S2. Forest plot of subgroup-specific hazard ratios for chemotherapy and overall survival from the main Cox model (Table S11). "Carcinoma (Ki-67 > 55%) before G3" denotes a documented Ki-67 >55% before baseline.

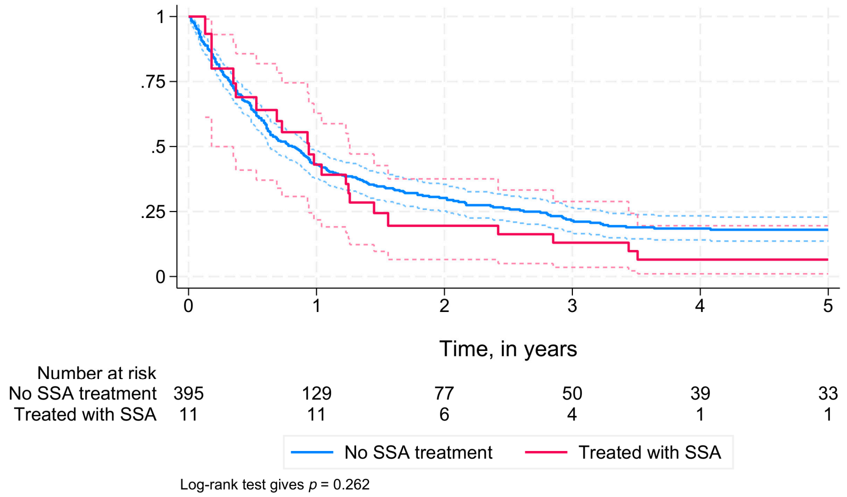

Figure S3. Kaplan-Meier curves for progression-free survival, comparing use and non-use of somatostatin analogues after exclusion of patients who started SSA before baseline. Log-rank p = 0.262. Because patients in the "ever used SSA" group cannot experience an event before starting SSA, a naive comparison of these curves is subject to immortal-time bias; the corresponding Cox model treats SSA use as a time-varying covariate (Table S9).

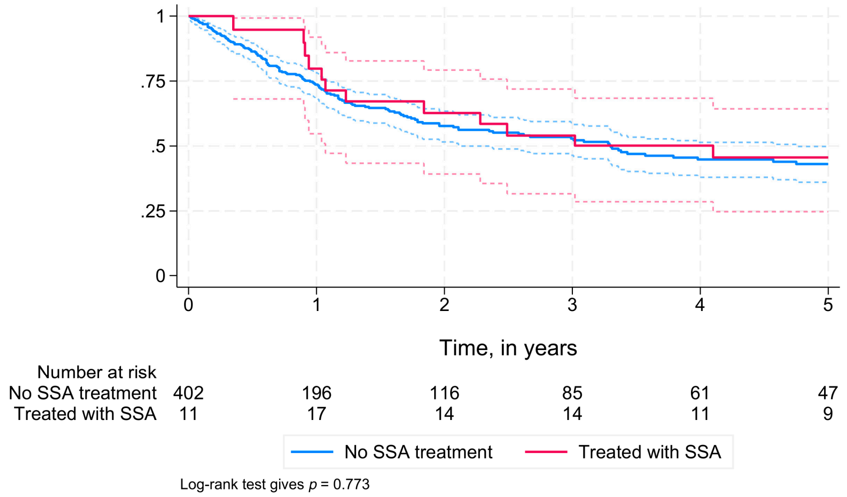

Figure S4. Kaplan-Meier curves for overall survival, comparing use and non-use of somatostatin analogues after exclusion of patients who started SSA before baseline. Log-rank p = 0.773 (Table S10).

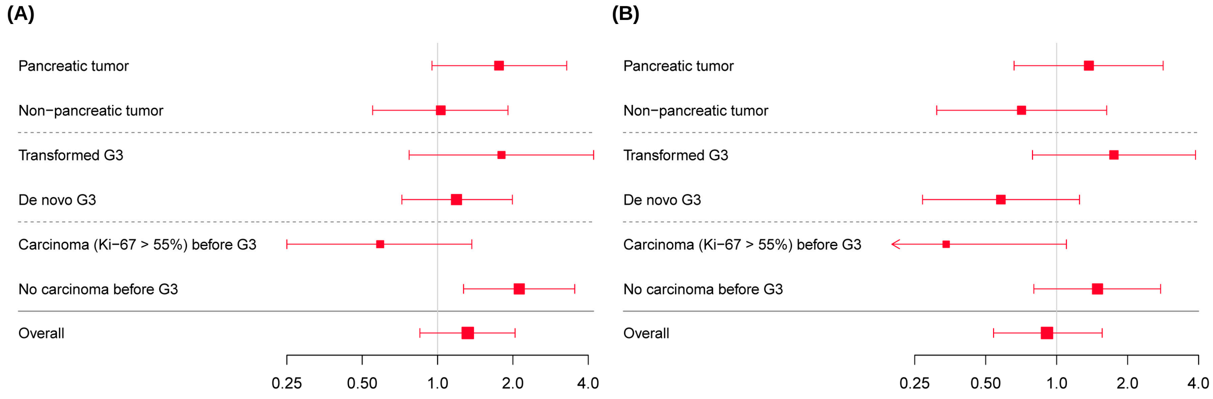

Figure S5. Forest plots of subgroup-specific hazard ratios for SSA use from the main Cox model (Table S12). (A) Progression-free survival. (B) Overall survival. “Carcinoma (Ki-67 > 55%) before G3” denotes a documented Ki-67 >55% before baseline.
